# Weighted gene co-expression network analysis identifies dysregulated B-cell receptor signaling pathway and novel genes in pulmonary arterial hypertension

**DOI:** 10.3389/fcvm.2022.909399

**Published:** 2022-10-06

**Authors:** Yuanrong Chen, Chaoling Wu, Xiaoping Wang, Xufeng Zhou, Kunpeng Kang, Zuofeng Cao, Yihong Yang, Yiming Zhong, Genfa Xiao

**Affiliations:** ^1^Key Laboratory of Prevention and Treatment of Cardiovascular and Cerebrovascular Diseases of Ministry of Education, Gannan Medical University, Ganzhou, China; ^2^Department of Cardiology, The First Affiliated Hospital of Gannan Medical University, Ganzhou, China; ^3^Gannan Branch Center of National Geriatric Disease Clinical Medical Research Center, Gannan Medical University, Ganzhou, China

**Keywords:** pulmonary arterial hypertension, B-cell receptor signaling pathway, weighted gene co-expression network analysis, humoral immunity, hub genes

## Abstract

**Background:**

Pulmonary arterial hypertension (PAH) is a devastating cardio-pulmonary vascular disease in which chronic elevated pulmonary arterial pressure and pulmonary vascular remodeling lead to right ventricular failure and premature death. However, the exact molecular mechanism causing PAH remains unclear.

**Methods:**

RNA sequencing was used to analyze the transcriptional profiling of controls and rats treated with monocrotaline (MCT) for 1, 2, 3, and 4 weeks. Weighted gene co-expression network analysis (WGCNA) was employed to identify the key modules associated with the severity of PAH. Gene Ontology (GO) and Kyoto Encyclopedia of Genes and Genomes (KEGG) enrichment analyses were performed to explore the potential biological processes and pathways of key modules. Real-time PCR and western blot analysis were used to validate the gene expression. The hub genes were validated by an independent dataset obtained from the Gene Expression Omnibus database.

**Results:**

A total of 26 gene modules were identified by WGCNA. Of these modules, two modules showed the highest correlation with the severity of PAH and were recognized as the key modules. GO analysis of key modules showed the dysregulated inflammation and immunity, particularly B-cell-mediated humoral immunity in MCT-induced PAH. KEGG pathway analysis showed the significant enrichment of the B-cell receptor signaling pathway in the key modules. Pathview analysis revealed the dysregulation of the B-cell receptor signaling pathway in detail. Moreover, a series of humoral immune response-associated genes, such as BTK, BAFFR, and TNFSF4, were found to be differentially expressed in PAH. Additionally, five genes, including BANK1, FOXF1, TLE1, CLEC4A1, and CLEC4A3, were identified and validated as the hub genes.

**Conclusion:**

This study identified the dysregulated B-cell receptor signaling pathway, as well as novel genes associated with humoral immune response in MCT-induced PAH, thereby providing a novel insight into the molecular mechanisms underlying inflammation and immunity and therapeutic targets for PAH.

## Introduction

Pulmonary arterial hypertension (PAH) is a devastating cardio-pulmonary vascular disease. Despite novel pharmacotherapy strategies improved symptoms and physical signs in patients with PAH, the prognosis remains poor (1). Occlusive and obliterative alterations in the small to medium pulmonary arteries are widely observed. Dysfunctional and altered structure in pulmonary vasculature leads to elevated mean pulmonary arterial pressure (mPAP), culminating in right ventricular failure and premature death (2). Dysregulated immunity and perivascular inflammation have attracted broad attention and have been inextricably associated with the development of PAH (2). Although there was accumulating research in PAH pathogenesis, with considerable progress in recent years, the exact molecular mechanism underlying inflammation and immunity in PAH remains unclear.

Monocrotaline (MCT), a plant-derived alkaloid, was extensively employed to induce PAH models for over 50 years (3). The development of MCT-induced PAH was closely associated with dysfunctional inflammatory and immune responses. In the MCT-induced PAH model, we found the thickened vascular wall, occlusive and/or obliterative vascular lumen, impaired endothelium, and perivasculitis in pulmonary arteries (4). Moreover, the parameters of pulmonary vascular remodeling, the percentage of total wall thickness to external diameters of pulmonary arterioles diameter (WT%), and the percentage of wall area to the total area of vessels (WA%) were gradually increased in the progression of MCT-induced PAH (4). In addition, we performed RNA sequencing (RNA-seq) analysis of MCT-induced PAH and found that inflammatory and immune responses occurred at the early stage of PAH and were involved in the initiation and progression of PAH (5).

The rapid development of systemic biology provides a powerful tool for exploring disease-associated genes (6). Weighted gene co-expression network analysis (WGCNA) is a novel systemic biology analysis approach (7) that divides the gene expression matrix into different gene modules and detects the relationship between gene modules and external phenotypical characteristics. WGCNA provides a system-level perspective into the genes and phenotypical traits and has been broadly used for probing the hub genes that drive key signaling pathways in diseases (8). For example, Hao et al. (9) identified specialized miRNA-mRNA regulatory networks composed of 6 miRNA and 12 mRNA in idiopathic pulmonary hypertension by using WGCNA. Zheng et al. (10) identified four key genes as potential therapeutic targets and early biomarkers in systemic sclerosis-related pulmonary hypertension by using WGCNA. In addition, Cai et al. (11) identified five immune cell-related marker genes by using WGCNA in pulmonary fibrosis-associated pulmonary hypertension, and these marker genes could divide these samples into various subgroups.

In this study, we constructed a gene co-expression network and identified the key modules related to the severity of PAH by performing WGCNA analysis, as well as characterized hub genes and signaling pathways, aiming to provide a deeper understanding of molecular mechanisms underlying inflammation and immunity in PAH.

## Materials and methods

### Data preparation

The detailed methods regarding animals and treatment, RNA extraction, cDNA library preparation, and RNA sequencing are described in our previous study (5, 12). Briefly, a total of 17 rats were used, in which 12 rats were randomly assigned to four MCT-treatment groups (*n* = 3, per group) and the remaining 5 rats served as a control group. The MCT-treated rats were sacrificed at the end of weeks 1, 2, 3, and 4. Five control rats were sacrificed at different time points, including week 0 (MCT was given) and each time when MCT-treated rats were sacrificed. Rat lungs were isolated for further RNA extraction, cDNA library preparation, and RNA-seq. The raw sequencing data have been deposited in Gene Expression Omnibus (GEO) with an accession number GSE149713. In addition, an independent dataset with an accession number GSE149899 was used to validate the hub genes. The raw read counts of the GSE149899 dataset were downloaded from GEO (https://www.ncbi.nlm.nih.gov/geo/query/acc.cgi?acc=GSE149899). The GSE149899 dataset included RNA-seq data of lung tissues from six irreversible PAH (MCT-induced PAH cannot be reversed by hemodynamic unloading) rats, 12 reversible PAH (MCT-induced PAH can be reversed by hemodynamic unloading) rats, and five normal control rats. The values of FPKM (fragments per kilobase of transcript per million fragments mapped) were usually used to estimate the relative expression abundance of transcripts. In the present study, the GSE149899 dataset was processed by transferring the raw read count into FPKM value in R software, so the gene expression value could be estimated and compared. The processed data of the GSE149899 dataset is attached in Supplementary Table 1.

### Construction of weighted gene co-expression network

In our previous study, a total of 23,200 transcripts were identified in MCT-induced PAH (5). To maintain data integrity, an expression matrix containing 17 samples and 23,200 transcripts was used to construct the co-expression network by using the “WGCNA” R package (7). First, the gene expression matrix was converted into the co-expression matrix by calculating the absolute value of each gene's correlation coefficient. Next, the co-expression matrix was transformed into the adjacency matrix by a thresholding procedure. Then, the “pickSoftThreshold” function of the “WGCNA” R package was used to perform an analysis of scale-free topology for soft-thresholding. Here, the soft-thresholding power β = 4 (scale-free *R*^2^ = 0.9) was chosen to meet the scale-free network. After that, the adjacency matrix was transformed into the topological overlap matrix (TOM) and dissimilarity TOM. Lastly, hierarchical clustering and the dynamic tree cut method were used to divide modules. The minimal gene number in each gene module was set as 50, and similar modules were merged based on a height cutoff of 0.25.

### Identification of key modules associated with PAH

Module eigengene (ME) is the first principal component of a given module and is considered a representative of the gene expression in the given module (7). ME was calculated for each module. The correlation between the MEs and the phenotypic traits was calculated by using the Pearson correlation coefficient to assess the relationship between each gene co-expression module and phenotypic trait. The phenotypic traits include WT%, WA%, mPAP, and right ventricular hypertrophy index (RVHI). These phenotypic traits were the parameters commonly used for assessing PAH severity (4). The modules showing the most highly positive or negative correlation with four phenotypic traits were selected as the key modules.

### Enrichment analysis of the key modules

Gene Ontology (GO) and Kyoto Encyclopedia of Genes and Genomes (KEGG) enrichment analysis were performed by the “clusterProfiler” R package (13). *P*-value < 0.05 was viewed as significant enrichment.

### Visualization of B-cell receptor signaling pathway

The genes annotated in the B-cell receptor (BCR) signaling pathway were extracted from KEGG PATHWAY DATABASE (https://www.genome.jp/kegg/pathway.html). The Pathview (https://pathview.uncc.edu/analysis) is a visual tool that maps, integrates, and renders a large variety of biological data onto molecular pathway graphs (14). The altered genes in the BCR signaling pathway were visualized by using a modified Pathview that showed *P*-value instead of expression value ratio change. The details of the statistical analysis are described in the figure legends.

### Identification of the hub genes

The genes with high connectivity in the key modules were viewed as the hub genes (7), which were considered to play a more significant biological role in the gene regulatory network. Module membership (MM) was used to represent intramodular connectivity by correlating the gene expression profile with the ME of a given module (15). The closer the MM of a gene is to 1 or −1, the higher the connection to the given module (7). Similarly, gene significance (GS) was used to assess the correlation between the gene expression profile and the phenotypic trait. The closer the GS of the gene is to 1, the more highly it is associated with the given phenotypic trait. In this study, genes with |GS| and |MM|≥ 0.8 were viewed as the candidate genes of the blue module, and genes with |GS|≥ 0.5 and |MM|≥ 0.8 were considered as the candidate genes of the dark red module. The differentially expressed genes (DEGs) in the candidate genes were defined as the hub genes in the present study. The hub genes were validated by GSE149899.

### Real-time PCR analysis

Total RNA was extracted from the lung tissues of control and rats injected with MCT for 4 weeks (MCTW4), according to the manufacturer's instruction. First-strand cDNA synthesis was performed by using the Transcriptor First Strand cDNA Synthesis Kit (Roche, Life Science, CH), according to the manufacturer's protocol. The quantification of mRNA expression was performed in a Step One Plus Real-time PCR System (Life Technologies™, Applied Biosystems, Gene Co., Ltd., Carlsbad, CA, USA). The rat origin primers used for real-time PCR analysis were listed as follows: forward-5′-ACA GCA ACA GGG TGG TGG AC-3′ and reverse-5′-TTT GAG GGT GCA GCG AAC TT-3′ for GAPDH; forward-5′-AGT CTG GTG GGC TGG AGG TGG-3′ and reverse-5′-GAA GGG TTT CCG AGG GGG GTA-3′ for BAFFR; forward-5′- ATG TGA GGG GGG AAG ACT A-3′ and reverse-5′- AGG TGG ATG AGA TAA AGC C-3′ for TNFSF4; forward-5′- CAA CAG CAG CAC CAA TCT CCA-3′ and reverse-5′-ATA CTC CTC GCC CTT TCG CAA-3′ for BTK; forward-5′-CTT GGG CAT TCT GTC GGT GAT-3′ and reverse-5′-GGA GGT GCT GGG AAG TTT ATT-3′ for CD20; and forward-5′-ATG GAC CAC CGC CTC TAC C-3′ and reverse-5′-TCC TCA GCC CCA CCA CAC A-3′ for IGHM. The relative quantification of the mRNA expression of genes was analyzed in accordance with the comparative 2^−ΔΔCT^ method and expressed as fold changes.

### Western blot analysis

Western blot analysis was performed as previously described (16). Lung tissues were isolated from the control group, and rats injected with MCT for 4 weeks. Then, the lung tissues were lysed with RIPA lysis buffer containing protease inhibitor cocktails (Meilunbio Biotech Co., Ltd, CHN). Tissue lysates were incubated on ice for 30 min and subsequently centrifuged at 12,000 rpm at 4°C for 5 min, and the supernatant was separated for further analysis. The protein concentration was measured by using the BCA kit (GBCBIO Biotech Co., Ltd, CHN). Equal amounts of total protein from different samples were separated by 12% SDS-PAGE and transferred into 0.45 μm PVDF membranes (IPVH00010, Merck Millipore, DEU). The PVDF membranes were blocked with 5% non-fat milk powder in TBST at room temperature for 2 h and incubated with primary antibodies overnight at 4°C. On the 2nd day, the membranes were washed three times with TBST and incubated with the appropriate horseradish peroxidase-conjugated secondary antibodies (Boster Biological Technology Co., Ltd, CHN, 1:10,000) for 2 h at room temperature. After being washed three times with TBST, the membranes were incubated with an ECL reagent (Affinity Biosciences, USA) for detecting the blots, and the blots were quantified by Image-Pro Plus software (version: 6.0). Primary antibodies, including mouse anti-BTK (1:1,000), rabbit anti-CD19 (1:1,000), rabbit anti-GAPDH (1:1,000), and rabbit anti-CD20 (1:2,000), were purchased from Jingjie PTM BioLab Co. Ltd, CHN.

### Statistical analysis

All statistical analyses were performed using RStudio (version 2021.09.2) and R (version 4.1.2) software. Data were presented as mean ± SD (standard deviation). The “ggplot2 (version 3.3.6),” “pheatmap (version 1.0.12)”, and “WGCNA (version 1.71)” R packages were used to draw graphics. The “DEseq2 (version 1.34.0)” R package was used to identify DEGs (a threshold of |log2 (Fold Change)| ≥1 & *p* ≤ 0.05). The difference between groups was compared by the two-tailed Student's *t*-test (normal distribution) or Wilcoxon signed-rank test (abnormal distribution). *P*-value < 0.05 was considered as statistical significance.

## Results

### Construction of the co-expression network and identification of the key modules

**Figure 1:**
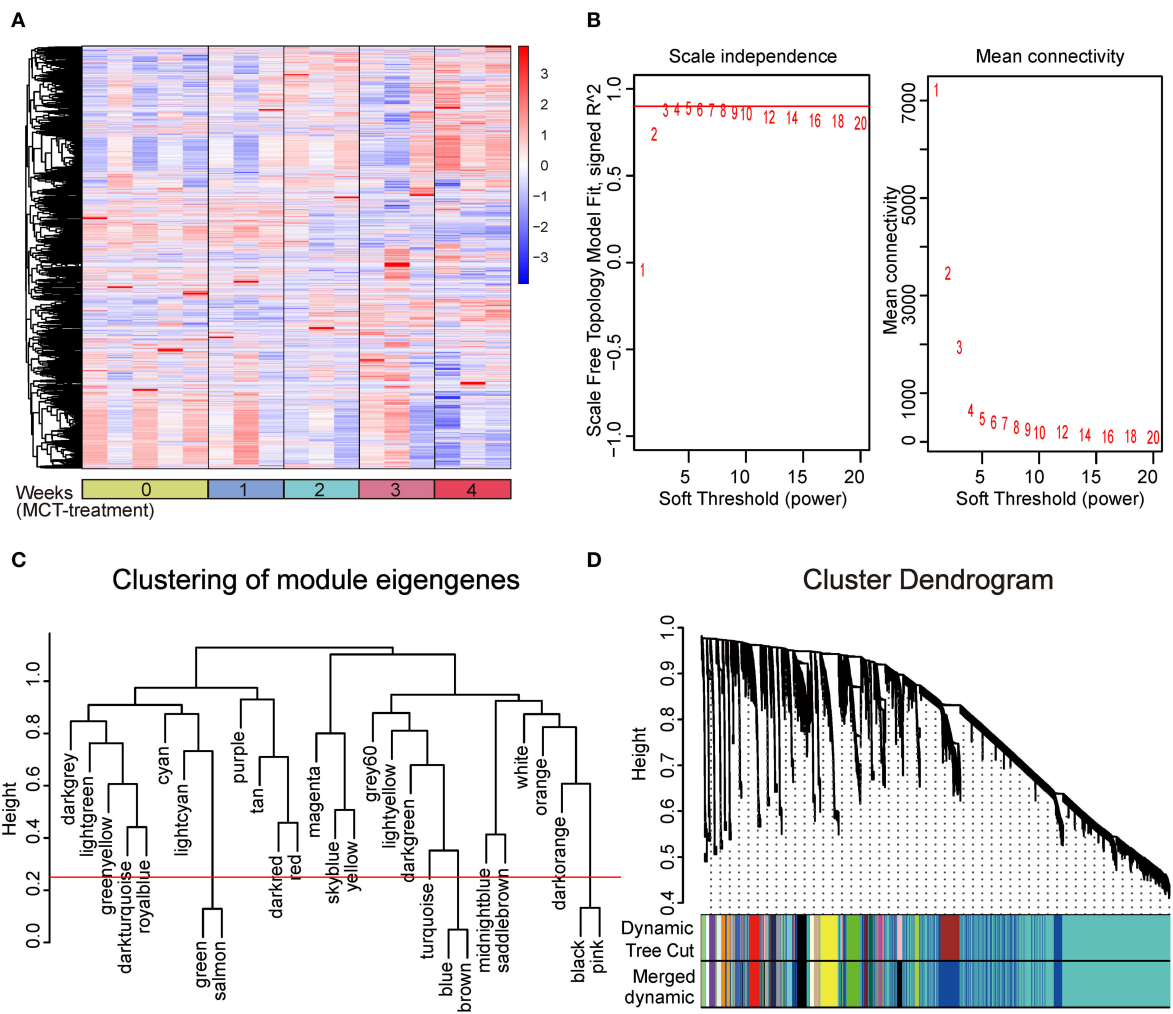
Construction of weighted gene co-expression network and division of gene co-expression module. **(A)** Clustering analysis heatmap plot of the expression matrix of 23,200 genes. The rows and columns represent gene expression levels and each sample, respectively. Red and blue represent the elevation and decrease of corresponding gene expression, respectively. **(B)** Analysis of the scale-free fit index (left) and the mean connectivity (right) for various soft-thresholding powers (β), among which four were chosen as the value to construct a scale-free network. **(C)** Clustering of module eigengenes. The cut height (red line) was 0.25. **(D)** Clustering dendrogram of all genes, with dissimilarity based on the topological overlap. Each color represents a gene co-expression module. Twenty-six gene co-expression modules were divided.

**Figure 2:**
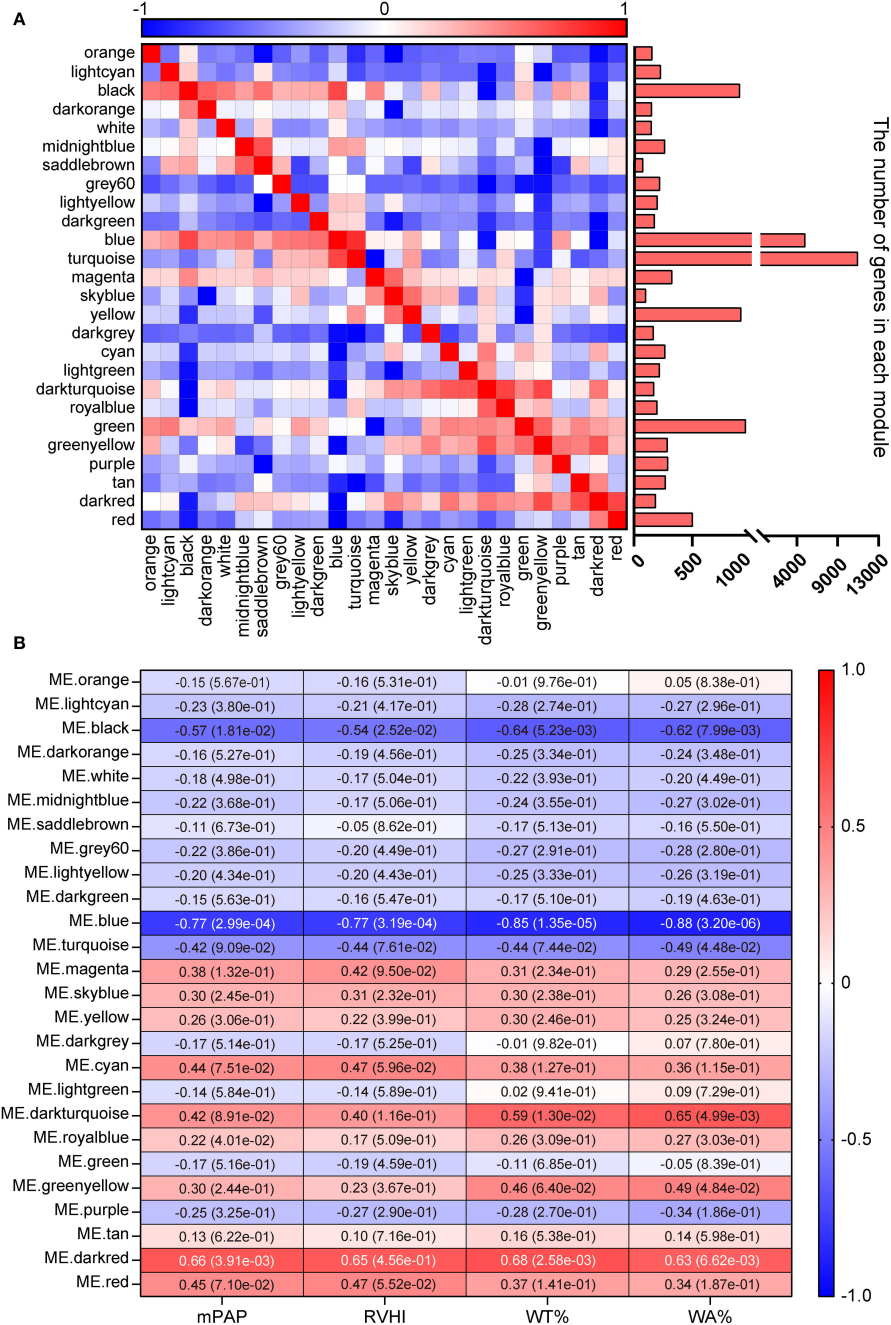
Identification of key modules. **(A)** Heatmap plot of module eigengene. Red and blue represent a positive and negative correlation, respectively. The bar chart represents the number of genes in each module. **(B)** Heatmap plot of module–trait relationship. Rows and columns represent different gene co-expression modules and phenotypic traits. The dark red and blue module showed the strongest correlation.

In this study, to ensure the integrity of transcriptional profiling, an expression matrix comprising 17 samples and 23,200 genes (Supplementary Table 2 and Figure 1A) was used to construct the co-expression network. Given the scale independence and mean connectivity, the power value β = 4 (scale-free *R*^2^ = 0.9) was selected as the soft threshold for the present study (Figure 1B). In addition, based on the dynamic tree cut algorithm and merge cutoff of 0.25, a total of 26 gene co-expression modules were generated and were assigned with different colors for distinguishing each other (Figures 1C,D). It was shown that the turquoise module had the largest number of genes, whereas the saddle brown showed the lowest number of genes (Figure 2A). The heatmap plot of ME showed that all gene modules were independent of each other (Figure 2A). The severity of MCT-induced PAH was evaluated by the phenotypic traits, composed of mPAP, RVHI, WT%, and WA% in our previous study (4). Upon constructing the co-expression network, we assessed the relationship between the gene co-expression modules and phenotypic traits through Pearson correlation analysis. As shown in Figure 2B, the dark red module showed the most highly positive correlation with mPAP (*r* = 0.66, *P* = 3.91e-03), RVHI (*r* = 0.65, *P* = 4.56e-01), WT% (*r* = 0.68, *P* = 2.58e-03), and WA% (*r* = 0.63, *P* = 6.62e-03). In contrast, the blue module showed the most highly negative correlation with mPAP (*r* = −0.77, *P* = 2.99e-04), RVHI (*r* = −0.77, *P* = 3.19e-04), WT% (*r* = −0.85, *P* = 1.35e-05), and WA% (*r* = −0.88, *P* = 3.20e-06). As a result, two modules depicted with blue and dark red colors were the most relevant to the severity of PAH and were identified as the key modules for further analysis.

### GO enrichment analysis of the key modules

**Figure 3:**
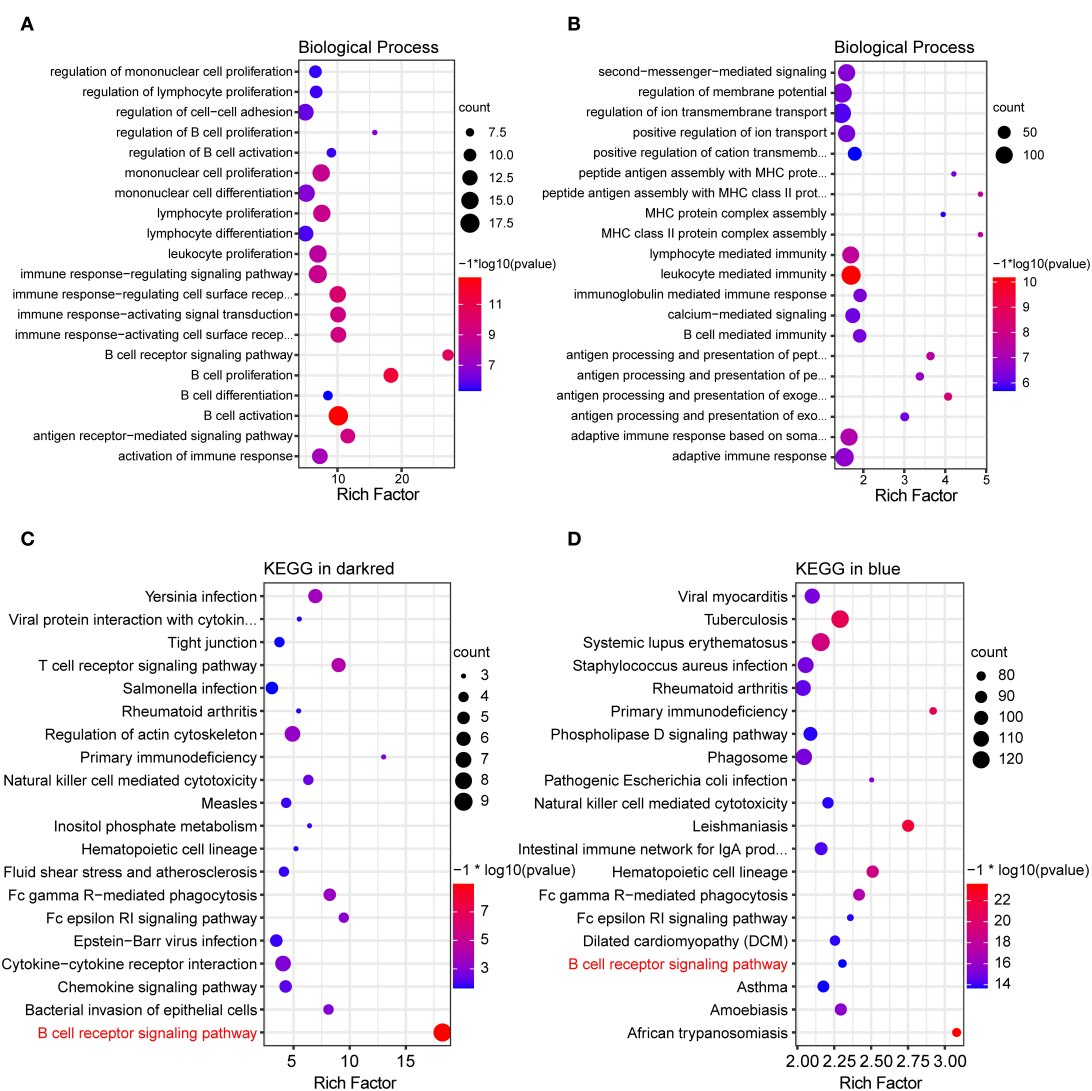
GO and KEGG functional enrichment analysis of key modules. **(A,B)** Bubble plot showing top 20 terms of biological process in the dark red and blue module, respectively. **(C,D)** Bubble plot showing top 20 pathways of KEGG functional enrichment analysis in the dark red and blue module, respectively.

To investigate potential biological processes of the dark red and blue module, GO enrichment analysis was performed. GO results of the dark red module showed that most of the biological processes were associated with humoral immune response, such as B-cell receptor signaling pathway, B-cell activation, B-cell proliferation, B-cell differentiation, antigen receptor-mediated signaling pathway, regulation of B-cell proliferation, and regulation of B-cell activation (Figure 3A). Similar to the dark red module, GO enrichment analysis of the blue module also showed a humoral immune response, such as antigen processing and presentation, B-cell-mediated immunity, and immunoglobulin-mediated immune response (Figure 3B). Taken together, these results suggested a role for B-cell-mediated humoral immune response in the development of MCT-induced PAH.

### KEGG enrichment analysis of the key modules

To further explore the inherent signaling pathways of the dark red and blue module, the KEGG enrichment analysis was performed. KEGG enrichment analysis of the dark red module revealed the inflammatory/immune-related pathways, including B-cell receptor (BCR) signaling pathway, T-cell receptor signaling pathway, primary immunodeficiency, chemokine pathway, natural killer cell-mediated cytotoxicity, and leukocyte trans-endothelial migration (Figure 3C). Consistently, the inflammatory/immune response-related pathways were also enriched in the blue module, owing to the enrichment of the BCR signaling pathway, primary immunodeficiency, and natural killer cell-mediated cytotoxicity (Figure 3D).

### The change of BCR signaling pathway in MCT-induced PAH

**Figure 4:**
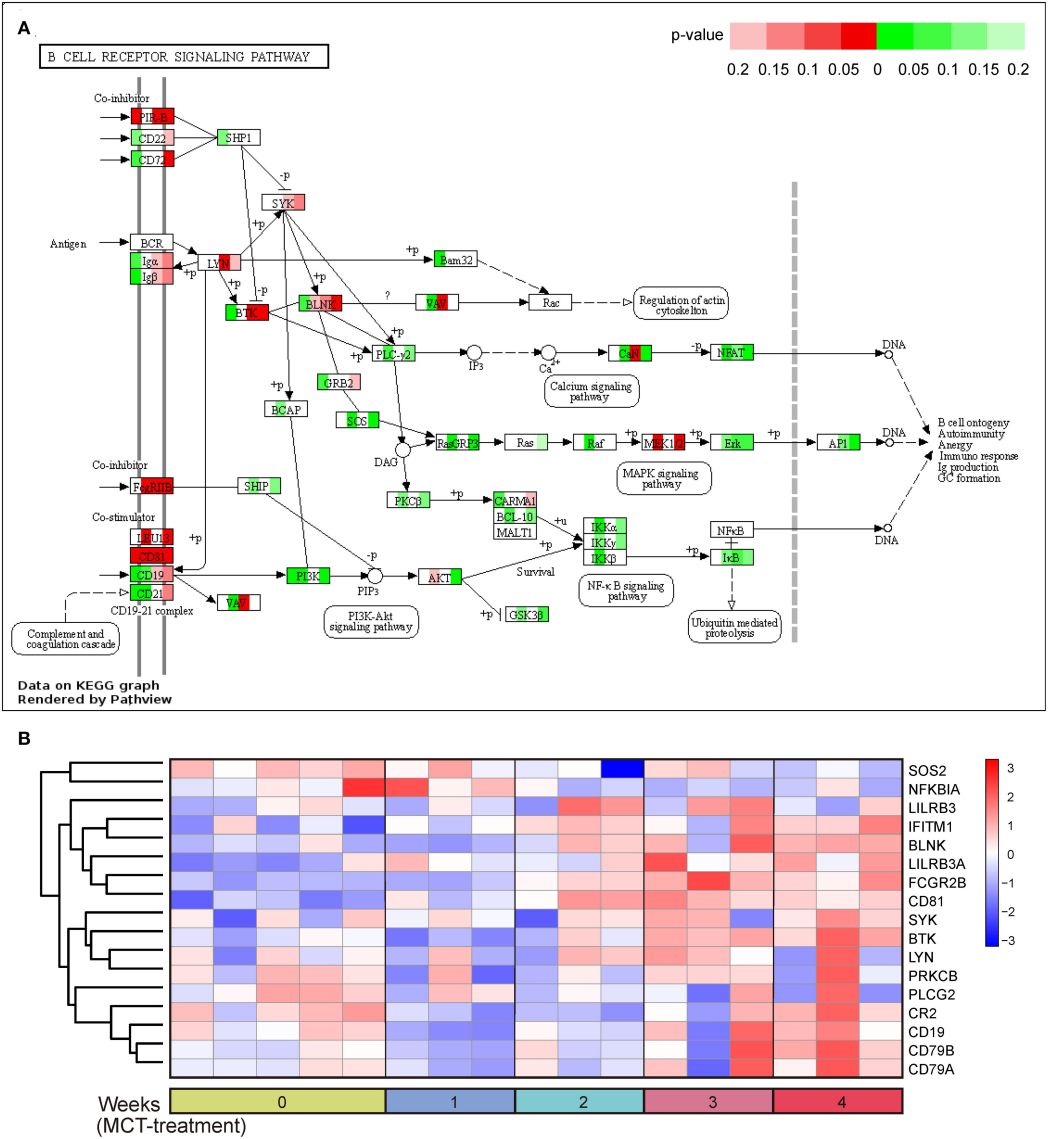
B-cell receptor signaling pathway in response to monocrotaline (MCT) treatment. **(A)** The integration and visualization of gene expression in B-cell receptor signaling pathway using modified Pathview. Each colored box represents the comparison of MCT treatment at week 1, 2, 3, and 4 with control, respectively. Color represents *P*-value for each comparison of MCT treatments with control (unpaired *t*-test), red and green represent the elevation and decrease of corresponding gene expression, respectively, while white represents *P* ≥ 0.2 or not detected. **(B)** Heatmap plot of differentially expressed genes in B-cell receptor signaling pathway. Rows and columns represent gene expression levels and each sample, respectively. Red and blue represent the elevation and decrease of corresponding gene expression, respectively.

It is worth noting that the BCR signaling pathway was the most significantly enriched pathway in the dark red module and was also significantly enriched in the blue module. Consequently, the BCR signaling pathway was chosen for further analysis. The BCR is an integral membrane complex composed of two immunoglobulin (Ig) heavy chains, two Ig light chains, and two heterodimers of Igα and Igβ (17). Ligation of the BCR with the specific antigen initiates highly sequential recruitment of critical intracellular mediators and adaptors, including LYN, SYK, BTK, BLNK, and VAV, and activates downstream pathways resulting in B-cell proliferation, differentiation, and secretion of various immunoglobulins and cytokines (18–20). Pathview and hierarchical clustering analysis showed the increased expression of BCR Igα (CD79A), Igβ (CD79B), LYN, BTK, SYK, BLNK, and CD19/21/81 in the BCR signaling pathway (Figures 4A,B). In addition, the altered expression of other genes was also shown, including upregulated genes FcgRIIB (FCGR2B), LEU13 (LFITM1), and PIR-B (LILRB3 and LILRB3A) and downregulated genes PLC-γ2 (PLCG2), PKCβ (PRKCB), SOS (SOS2), and IκBα (NFKBIA).

### Identification and validation of humoral immune response-associated DEGs

**Figure 5:**
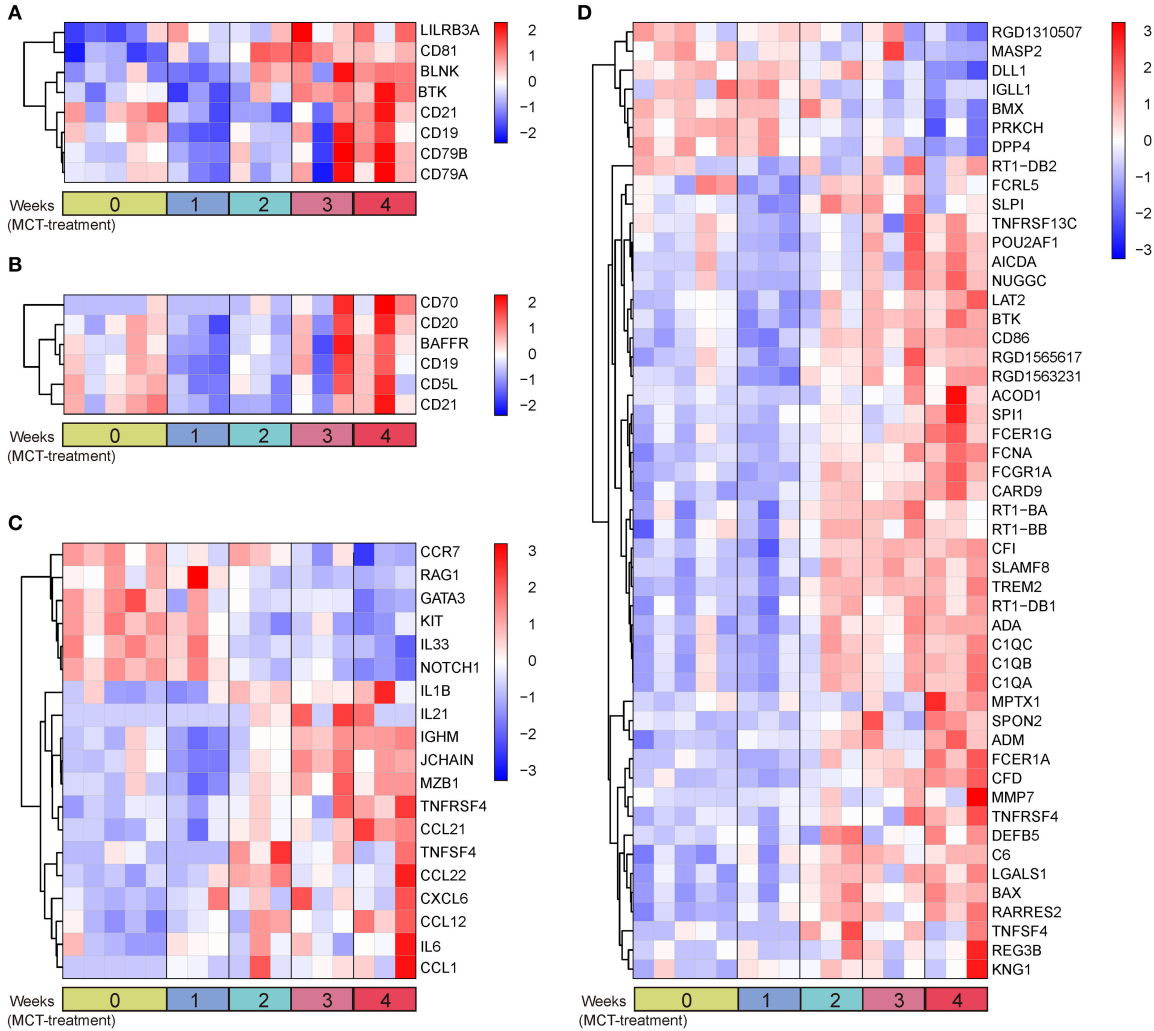
Heatmap plot of humoral immune response-associated DEGs. **(A)** Eight overlapped DEGs in GO terms and BCR signaling pathway. **(B)** DEGs in B-cell homeostasis (GO:0001782), activation (GO:0042113), and proliferation (GO:0042100). **(C)** DEGs in immature B-cell differentiation (GO:0002327), immunoglobulin production (GO:0002377), and humoral immune response (GO:0006959). **(D)** DEGs in remaining unassorted terms associated with humoral immune response. Rows and columns represent gene expression levels and each sample, respectively. Red and blue represent the elevation and decrease of corresponding gene expression, respectively.

Due to the enrichment of dysregulated BCR signaling pathway, we then analyzed the genes enriched in the humoral immune response by GO analysis. A total of 26 terms and 162 genes were associated with humoral immune response in the dark red and blue module (Supplementary Table 3). Differential expression analysis showed that 78 genes were differentially expressed, of which eight genes, including CD19, CD21, CD81, BTK, BLNK, CD79A, CD79B, and LILRB3A, were overlapped in the BCR signaling pathway (Figure 5A). To characterize these humoral immune response-associated DEGs, we applied a heatmap to exhibit them. The heatmap showed that the majority of genes were elevated in a time-dependent manner (Figures 5A–D). DEGs associated with cellular B-cell homeostasis (GO:0001782), activation (GO:0042113), and proliferation (GO:0042100) were upregulated in MCT-induced PAH, such as BAFFR, CD70, and CD20 (Figure 5B). In contrast, DEGs associated with immature B-cell differentiation (GO:0002327), immunoglobulin production, (GO:0002377), and humoral immune response (GO:0006959) showed dysregulated expression, due to the identification of both upregulated DEGs (IL-1β, IL-6, IGHM, JCHAIN, TNFSF4, TNFRSF4, and MZB1) and downregulated DEGs (IL-33, NOTCH1, CCR7, etc.) (Figure 5C). As shown in Figure 5D, the upregulation of DEGs associated with the regulation of B-cell activation (GO:0050864), proliferation (GO:0030888), differentiation (GO:0030183), humoral immune response (GO:0002920), and BCR signaling pathway (GO:0050855) was also identified, such as ACOD1 and FCRL5. Moreover, a set of DEGs was overlapped in various biological process terms. For instance, BTK was overlapped in B-cell-mediated immunity (GO:0019724), B-cell receptor signaling pathway (GO:0050853), and positive regulation of B-cell-mediated immunity (GO:0002714). BAFFR was overlapped in B-cell homeostasis (GO:0001782), activation (GO:0042113), proliferation (GO:0042100), and regulation of B-cell activation (GO:0050864) and proliferation (GO:0030888). TNFSF4 was overlapped in B-cell-mediated immunity (GO:0019724), immunoglobulin production (GO:0002377), and positive regulation of B-cell-mediated immunity (GO:0002714).

**Figure 6:**
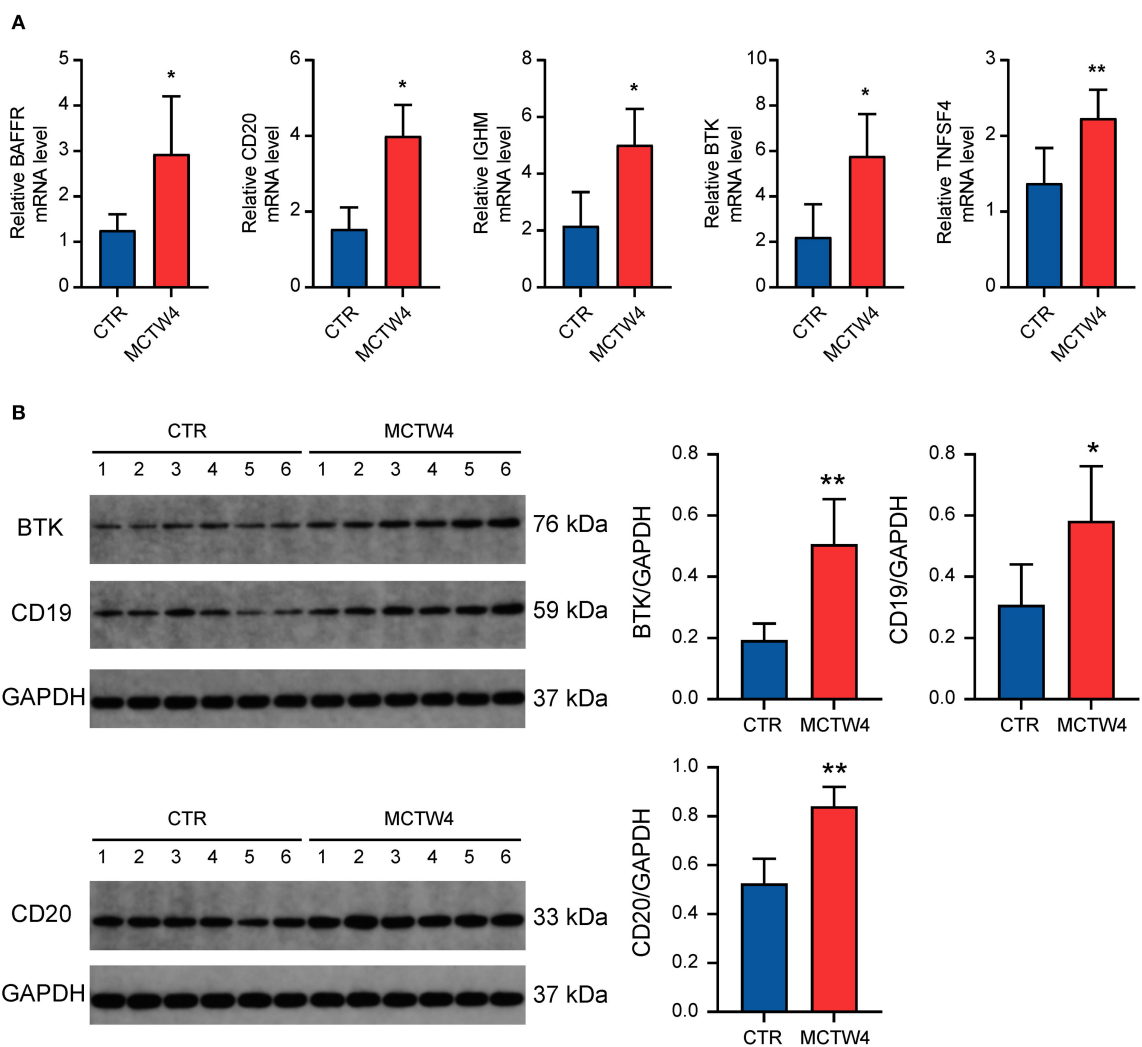
Validation of the expression of critical humoral immune response-associated genes. **(A)** Real-time PCR analysis. Data were shown as mean ± SD. *n* = 3 MCT-treated rats at 4 weeks, and *n* = 5 control. ^*^P < 0.05 vs control; ^**^ *P* < 0.01 vs. control. **(B)** Western blot analysis. Data are shown as mean ± SD. *n* = 6 MCT-treated rats at week 4, and *n* = 6 control. ^*^P < 0.05 vs. control; ^**^ *P* < 0.01 vs. control. CTR, control; MCTW4, MCT treatment for 4 weeks.

The DEGs overlapped in multiple GO terms may be more important and representative of humoral immunity. Therefore, we selected BAFFR, BTK, and TNFSF4 for further validation. Additionally, CD19, CD20, and IGHM were also selected, due to their essential roles in humoral immune response (21, 22). As shown in Figure 6A, the validation of the RNA-seq dataset by real-time PCR showed the upregulation of BAFFR, BTK, TNFSF4, CD20, and IGHM, thus confirming the upregulation of humoral immune response-associated genes in MCT-induced PAH. To further validate our RNA-seq dataset in protein levels, western blot analysis was performed to validate three critical humoral immune response-associated DEGs, including BTK, CD19, and CD20. Western blot analysis showed elevated protein levels of BTK, CD19, and CD20 (Figure 6B), further confirming the upregulation of humoral immune response-associated genes in MCT-induced PAH.

### Identification and validation of the hub genes in key modules

**Figure 7:**
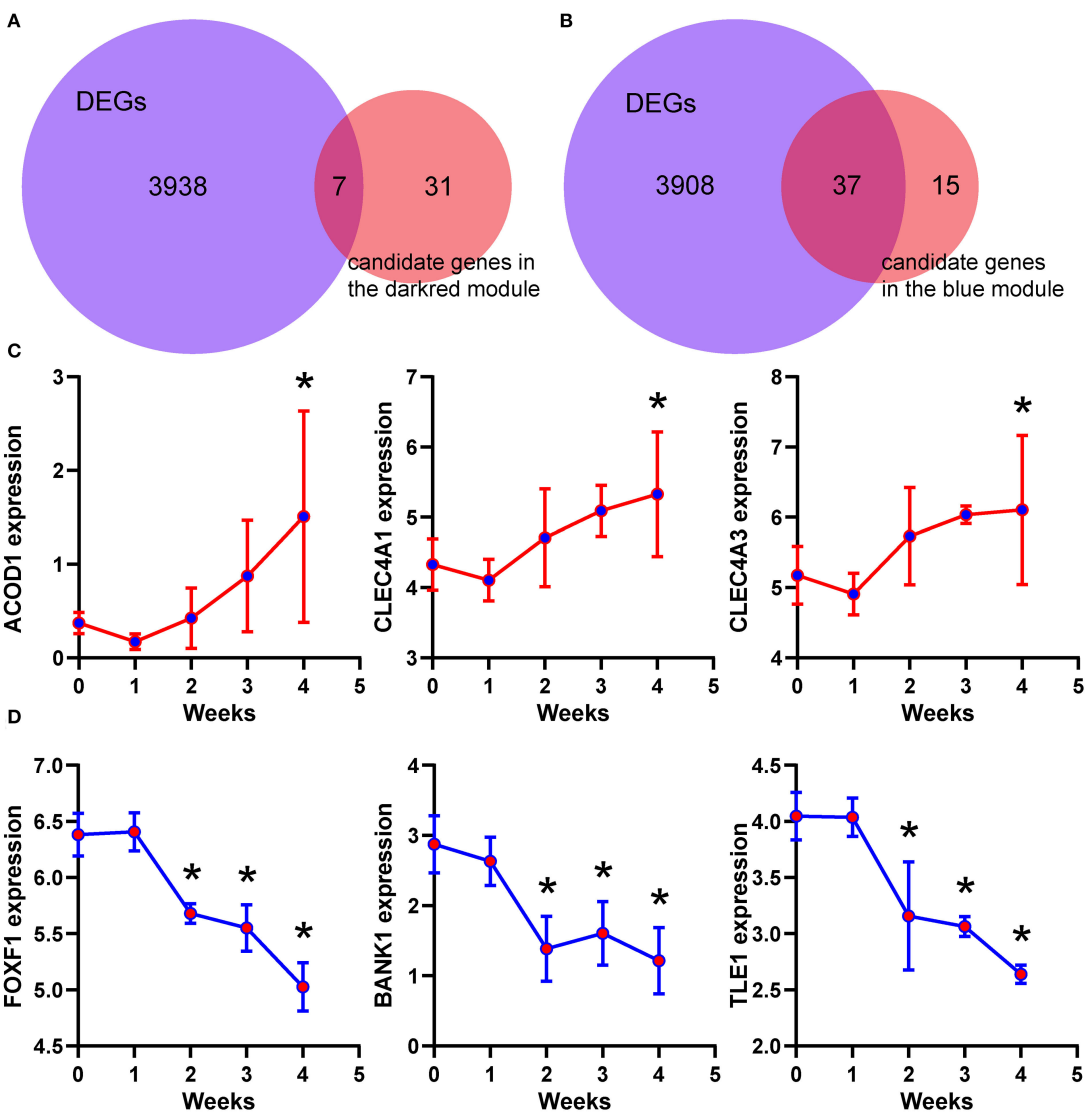
Identification of the hub genes. **(A,B)** Venn diagram showing the overlapped gene numbers between differentially expressed genes and candidate genes. **(C,D)** Temporal expression levels of six hub genes. Data are shown as mean ± S.E.M. *n* = 3 MCT-treated rats at each week, and *n* = 5 control. ^*^P < 0.05 vs. control.

**Figure 8:**
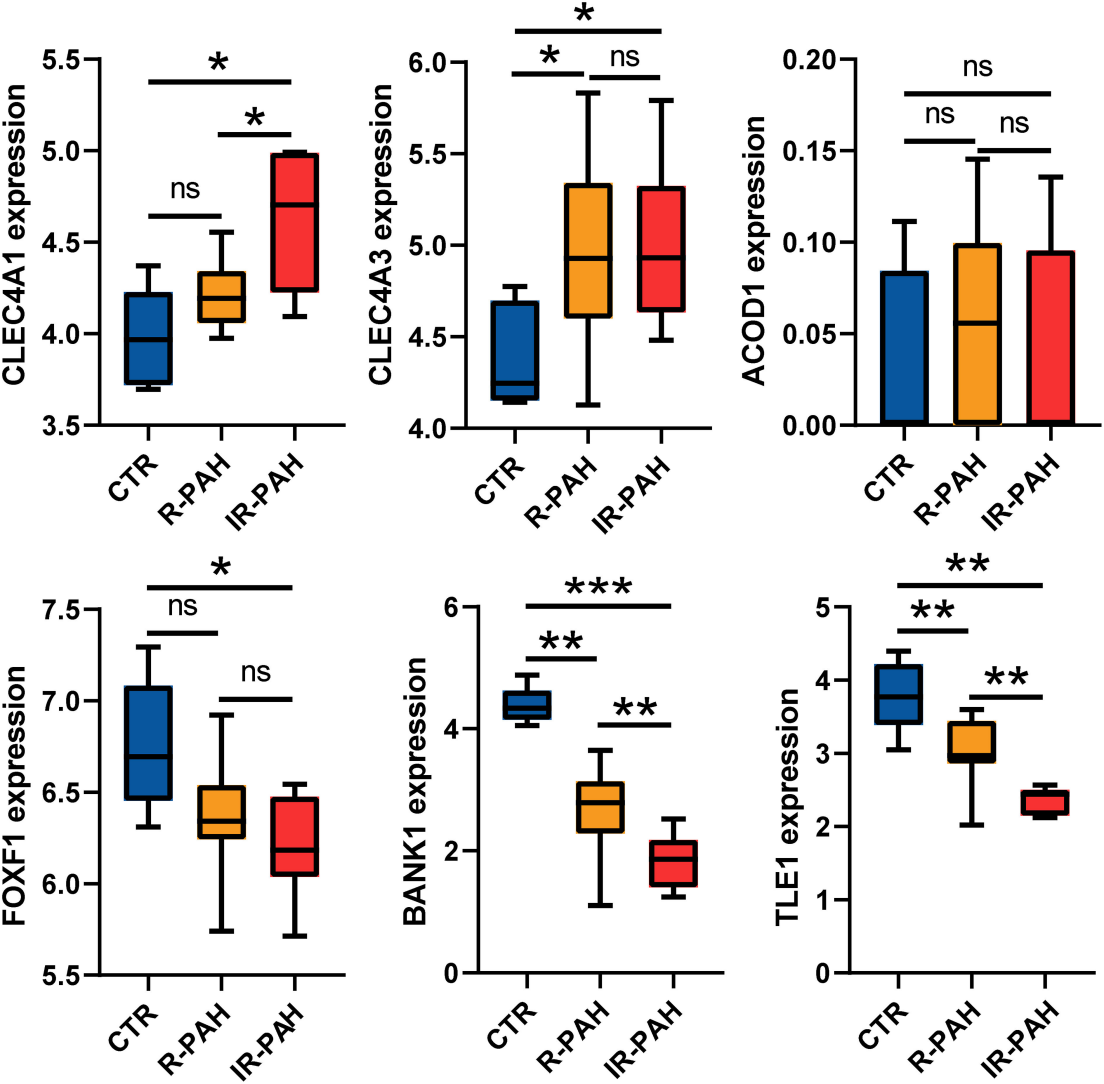
Validation of the hub genes. Boxplots showing the expression level of six real hub genes in dataset GSE149899. ^*^P < 0.05, ^**^ *P* < 0.01, ^***^ *P* < 0.001, ns, no significance. CTR, control; R-PAH, reversible pulmonary arterial hypertension; IR-PAH, irreversible pulmonary arterial hypertension.

According to the criteria mentioned in the methods, 38 and 52 candidate genes were identified in the dark red and blue module, respectively. Differential expression analysis showed that 7 of 38 candidate genes, including ACOD1, CLEC4A1, and CLEC4A3, were differentially expressed in the dark red module (Figure 7A and Supplementary Figure 1). Similarly, 37 of 52 candidate genes, including FOXF1, BANK1, and TLE1, were differentially expressed in the blue module (Figure 7B and Supplementary Figure 2). As shown in Figure 7C, the expression of the ACOD1, CLEC4A1, and CLEC4A3 was gradually increased in the dark red module. In contrast, the expression of the FOXF1, BANK1, and TLE1 was gradually reduced in the blue module (Figure 7D). We then confirmed the expression of these hub genes by the GSE149899 dataset. As shown in Figure 8, in addition to ACOD1, the expression of CLEC4A1 and CLEC4A3 was increased, while that of FOXF1, BANK1, and TLE1 was reduced.

## Discussion

In this study, WGCNA was used to identify pathways and hub genes related to the severity of PAH induced by MCT. We found the dysregulated BCR signaling pathway and humoral immune response-associated genes in MCT-induced PAH.

We identified a total of 26 gene co-expression modules that were independent of each other. Further analysis was based on the module–trait relationships. Because the blue and dark red module showed the strongest correlation with the phenotypic traits that reflected the severity of PAH, we selected the dark red and blue module as the key modules, and further enrichment analysis was carried out. GO enrichment analysis showed that the genes in key modules were mainly associated with B-cell-mediated humoral response, including B-cell activation, regulation of B-cell activation, positive regulation of B-cell activation, and immunoglobulin production. Moreover, KEGG enrichment analysis showed that the BCR signaling pathway was enriched in both the dark red and blue module. Then, we used Pathview analysis to show the detailed changes in the BCR signaling pathway in MCT-induced PAH. As it was exhibited by Pathview, visualization of global pathway changes showed that the gene expression in the BCR signaling pathway was not synergistic. For instance, the decreased expression of PLC-γ2, PKCβ, and IκBα was identified in the BCR signaling pathway. The decreased levels of IκBα, an inhibitor of NF-κB, activates NF-κB signaling pathway, whereas the reduced levels of PLC-γ2 and PKCβ may limit the activation of the NF-κB signaling pathway. Therefore, it was possible that the presence of the negative feedback mechanisms restricted the over activated or prolonged BCR signaling pathway in the progression of PAH. The humoral immune response-associated GO terms and BCR signaling have already been enriched in our previous study (5, 12). As a result, the present study may extend and expand B-cell-mediated humoral immunity in the development of PAH.

It is well-established that B cells are essential to humoral immune response and are associated with autoimmune diseases (23). The B-cell-mediated humoral immune response involves a series of processes in which, upon recognition of the specific antigen, B cells are activated, and the activated B cells proliferate to form germinal centers and differentiate into plasma cells to produce antibodies (24). In the present study, a series of B-cell markers, including CD19, CD20, CD79A, and CD79B (25, 26), were elevated. The identification of increased B-cell markers indicated elevated B-cell infiltration in the lung tissues of MCT-induced PAH, which was consistent with current pathological findings (27, 28). Moreover, reduced CCR7 level was identified; of note, the lack of CCR7 resulted in the infiltration of perivascular lymphocytes, including B cells, in pulmonary hypertension (29). Furthermore, some of these B-cell markers were considered as potential targets of B-cell depletion therapy in PAH. For example, rituximab, an anti-CD20 B-cell antibody, had shown accepted safety and tolerability for the treatment of PAH with systemic sclerosis or systemic lupus erythematosus (30, 31).

A set of genes associated with humoral immune response were upregulated, including BAFFR, TNFSF4, TNFRSF4, MZB1, BTK, CD19, and immunoglobulin chain IGHM and JCHAIN. The BAFFR is indispensable for maintaining the development, maturation, and survival of B cells (32). Moreover, the deficiency of BAFFR in either mice or humans results in decreased titers of serum IgM (33, 34). The interaction between TNFSF4 and TNFRSF4 is necessary for differentiating activated B cells into plasma cells and producing antibodies (35). TNFSF4 promoted the affinity maturation of the secondary humoral immune response in B cells, and the knockout of TNFSF4 generated a reduced proportion of germinal center B cells and plasma cells (36). Moreover, a lack of TNFSF4 ameliorated the autoimmune phenotype and reduced the total IgM (36). MZB1 regulates B cells to produce IgM antibodies, and downregulation or deficiency of MZB1 led to impaired IgM secretion (37). CD19 acts as the costimulatory molecule, and plays a crucial role in regulating B-cell-mediated humoral immune response. The deficiency of CD19 severely impaired B-cell maturation and germinal center formation, accompanied by a lack of B cells and IgM (21, 22). In contrast, hCD19 transgenic mice showed significantly spontaneous B-cell proliferation and an overall elevated level of immunoglobulins, including IgM (21). Interestingly, the expression of BAFFR, TNFSF4, TNFRSF4, MZB1, CD19, IGHM, and JCHAIN was elevated in the present study, indicating that B cells were activated to produce antibodies, thus in line with the evidence of local immunoglobulin production in PAH (38). It is noteworthy that activated B cells not only produce antibodies but also secrete a variety of cytokines involved in the immune response, such as IL-1β and IL-6 (20, 39). The identification of elevated cytokines IL-1β and IL-6 suggested enhanced B-cell-mediated antibody-independent functions. BTK is a crucial protein in BCR signaling, and increased BTK levels are associated with autoimmune diseases (40). The overexpression of BTK-induced spontaneous germinal center formation and production of autoantibodies in CD19-hBTK transgenic mice (41), and moreover, induced pulmonary hypertension through activation of BCR signaling in pulmonary injury mice (42). The BTK expression was upregulated in the present study, further supporting the role of the dysregulated BCR signaling pathway in MCT-induced PAH.

BANK1 is a scaffold protein that is primarily expressed in B lineage cells (43). Functional variant and polymorphism of BANK1 are associated with susceptibility to autoimmune diseases, such as systemic lupus erythematosus (44), rheumatoid arthritis (45), diffuse cutaneous systemic sclerosis (46), and primary Sjogren's syndrome (47), suggesting an important role of BANK1 in autoimmune diseases. Upregulated BANK1 expression was mainly observed in immune tolerance patients (48). By contrast, decreased BANK1 expression was observed in activated B cells and plasma cells (48). It was reported that decreased BANK1 exacerbated B-cell response, resulting in the elevation of autoantibodies in collagen-induced arthritis mice (49) and also augmented germinal center formation and IgM production in BANK1-deficient mice (50). The reduced BANK1 was identified and validated in the present study. It was possible that the downregulation of BANK1 contributed to the loss of immune tolerance in patients and susceptibility to PAH.

FOXF1 is a transcription factor that plays an essential role in lung development (51). FOXF1 regulates pulmonary angiogenesis by regulating STAT3 (52) and VEGF (53, 54) signaling in endothelial cells. The deletion of heterozygous copy number variants and point mutations in FOXF1 is the primary cause of alveolar capillary dysplasia with misalignment of pulmonary veins (55, 56), which is a rare and fatal disease characterized by severe progressive hypoxia and pulmonary hypertension (57). The identification of reduced FOXF1 may partially explain angiogenesis deficiency in pulmonary hypertension.

TLE1 is a corepressor that regulates the transcription of downstream genes by interacting with transcription factors and other proteins (58). Overexpression of TLE1 causes a decrease in nuclear NF-κB translocation, and the deficiency of TLE1 enhanced inflammatory responses mediated by Toll-like receptors (59). Moreover, TLE1 regulates NOD2/NF-κB signaling to mediate inflammatory responses (60, 61). Interestingly, we have reported that dysregulated Toll-like receptor and Nod-like receptor pathways lead to inflammatory cell infiltration and pulmonary vascular remodeling in PAH (12). Thus, TLE1 may act as a negative regulator involved in Toll-like and Nod-like receptor pathways in PAH.

CLEC4A1 and CLEC4A3 are members of the transmembrane C-type lectin receptors (CLRs). CLRs are expressed in various cells, including dendritic cells, B cells, monocytes, and macrophages (62). Damage-associated molecular patterns (DAMPs) can initiate CLR-mediated inflammatory responses that have been linked to allergy (63). We have previously demonstrated that, in MCT-induced PAH, the RIPK3-mediated necroptosis released a series of DAMPs (12). It is likely that CLR-mediated inflammatory responses triggered by DAMPs also participated in the PAH pathogenesis.

Several limitations are present in this study. First, the biological replicates in each MCT treatment group may be limited. Second, the results in this study were based on transcriptomic data, and the protein levels remain unknown. Thus, further studies are needed to validate our results.

## Conclusion

In this study, WGCNA was employed to analyze the transcriptome of MCT-induced PAH for the first time. We identify dysregulated BCR signaling pathways and novel genes related to humoral immune response. Thus, these results may provide a novel insight into the molecular mechanisms underlying inflammation and immunity and therapeutic targets for PAH.

## Data availability statement

The datasets presented in this study can be found in online repositories. The names of the repository/repositories and accession number(s) can be found below: https://www.ncbi.nlm.nih.gov/geo/, GSE149713, GSE149899 public in 2020.

## Author contributions

YC and GX conducted this study. YC wrote the first draft of the manuscript. GX conceptualized this study. GX and YZ revised this manuscript. CW, XW, XZ, ZC, YY, and YZ were responsible for the literature search and reviewed the manuscript. All authors contributed to the article and approved the final version.

## Funding

This work was supported by the Open Project of Key Laboratory of Prevention and Treatment of Cardiovascular and Cerebrovascular Disease, Ministry of Education (Grant No. XN202010 to GX) and the Graduate Student Innovation Special Foundation of Jiangxi Province of China (Grant No. YC2021-S800 to YC).

## Conflict of interest

The authors declare that the research was conducted in the absence of any commercial or financial relationships that could be construed as a potential conflict of interest.

## Publisher's note

All claims expressed in this article are solely those of the authors and do not necessarily represent those of their affiliated organizations, or those of the publisher, the editors and the reviewers. Any product that may be evaluated in this article, or claim that may be made by its manufacturer, is not guaranteed or endorsed by the publisher.

## Supplementary material

Supplementary Figure 1 Heatmap of seven hub genes in the dark red module.

Supplementary Figure 2 Heatmap of 37 hub genes in the blue module.

Supplementary Table 1 A matrix of GSE149899 dataset.

Supplementary Table 2 A matrix of 23,200 transcripts.

Supplementary Table 3 Twenty-six biological processes related to humoral immune response.

## References

[1] Benza RL Miller DP Gomberg-Maitland M Frantz RP Foreman AJ Coffey CS. Predicting survival in pulmonary arterial hypertension: insights from the registry to evaluate early and long-term pulmonary arterial hypertension disease management (Reveal). Circulation. (2010) 122:164–72. 10.1161/CIRCULATIONAHA.109.898122 20585012

[2] Rabinovitch M Guignabert C Humbert M Nicolls MR. Inflammation and immunity in the pathogenesis of pulmonary arterial hypertension. Circ Res. (2014) 115:165–75. 10.1161/CIRCRESAHA.113.301141 24951765 PMC4097142

[3] Hill NS Gillespie MN McMurtry IF. 50 years of monocrotaline-induced pulmonary hypertension: what has it meant to the field? Chest. (2017) 152:1106–8. 10.1016/j.chest.2017.10.007 29223258

[4] Zhuang W Lian G Huang B Du A Xiao G Gong J. Pulmonary arterial hypertension induced by a novel method: twice-intraperitoneal injection of monocrotaline. Exp Biol Med. (2018) 243:995–1003. 10.1177/1535370218794128 30099957 PMC6180403

[5] Xiao G Wang T Zhuang W Ye C Luo L Wang H. RNA sequencing analysis of monocrotaline-induced pah reveals dysregulated chemokine and neuroactive ligand receptor pathways. Aging. (2020) 12:4953–69. 10.18632/aging.102922 32176619 PMC7138548

[6] Zupanic A Bernstein HC Heiland I. Systems biology: current status and challenges. Cell Mol Life Sci. (2020) 77:379–80. 10.1007/s00018-019-03410-z 31932855 PMC11104875

[7] Langfelder P Horvath S. Wgcna: an r package for weighted correlation network analysis. BMC Bioinformatics. (2008) 9:559. 10.1186/1471-2105-9-559 19114008 PMC2631488

[8] Liu W Li L Ye H Tu W. Weighted gene co-expression network analysis in biomedicine research. Sheng Wu Gong Cheng Xue Bao. (2017) 33:1791–801. 10.13345/j.cjb.170006 29202516

[9] Hao S Jiang P Xie L Xiang G Liu Z Hu W. Essential genes and mirna-mrna network contributing to the pathogenesis of idiopathic pulmonary arterial hypertension. Front Cardiovasc Med. (2021) 8:627873. 10.3389/fcvm.2021.627873 34026864 PMC8133434

[10] Zheng JN Li Y Yan YM Shi H Zou TT Shao WQ. Identification and validation of key genes associated with systemic sclerosis-related pulmonary hypertension. Front Genet. (2020) 11:816. 10.3389/fgene.2020.00816 32793290 PMC7393672

[11] Cai H Liu H. Immune infiltration landscape and immune-marker molecular typing of pulmonary fibrosis with pulmonary hypertension. BMC Pulm Med. (2021) 21:383. 10.1186/s12890-021-01758-2 34823498 PMC8614041

[12] Xiao G Zhuang W Wang T Lian G Luo L Ye C. Transcriptomic analysis identifies toll-like and nod-like pathways and necroptosis in pulmonary arterial hypertension. J Cell Mol Med. (2020) 24:11409–21. 10.1111/jcmm.15745 32860486 PMC7576255

[13] Yu G Wang LG Han Y He QY. Clusterprofiler: an R package for comparing biological themes among gene clusters. Omics. (2012) 16:284–7. 10.1089/omi.2011.0118 22455463 PMC3339379

[14] Luo W Pant G Bhavnasi YK Blanchard SG Jr Brouwer C. Pathview web: user friendly pathway visualization and data integration. Nucleic Acids Res. (2017) 45:W501–w8. 10.1093/nar/gkx372 28482075 PMC5570256

[15] Bai Z Xu L Dai Y Yuan Q Zhou Z. Ecm2 and Glt8d2 in human pulmonary artery hypertension: fruits from weighted gene co-expression network analysis. J Thorac Dis. (2021) 13:2242–54. 10.21037/jtd-20-3069 34012575 PMC8107565

[16] Xiao G Lian G Wang T Chen W Zhuang W Luo L. Zinc-mediated activation of creb pathway in proliferation of pulmonary artery smooth muscle cells in pulmonary hypertension. Cell Commun Signal. (2021) 19:103. 10.1186/s12964-021-00779-y 34635097 PMC8504081

[17] Treanor B. B-cell receptor: from resting state to activate. Immunology. (2012) 136:21–7. 10.1111/j.1365-2567.2012.03564.x 22269039 PMC3372753

[18] Harwood NE Batista FD. Early events in B cell activation. Annu Rev Immunol. (2010) 28:185–210. 10.1146/annurev-immunol-030409-101216 20192804

[19] Siebenlist U Brown K Claudio E. Control of lymphocyte development by nuclear factor-Kappab. Nat Rev Immunol. (2005) 5:435–45. 10.1038/nri1629 15905862

[20] Shen P Fillatreau S. Antibody-independent functions of B cells: a focus on cytokines. Nat Rev Immunol. (2015) 15:441–51. 10.1038/nri3857 26065586

[21] Engel P Zhou LJ Ord DC Sato S Koller B Tedder TF. Abnormal B lymphocyte development, activation, and differentiation in mice that lack or overexpress the Cd19 signal transduction molecule. Immunity. (1995) 3:39–50. 10.1016/1074-7613(95)90157-4 7542548

[22] Rickert RC Rajewsky K Roes J. Impairment of T-cell-dependent B-cell responses and B-1 Cell Development in Cd19-Deficient Mice. Nature. (1995) 376:352–5. 10.1038/376352a0 7543183

[23] Khan WN Wright JA Kleiman E Boucher JC Castro I Clark ES. B-lymphocyte tolerance and effector function in immunity and autoimmunity. Immunol Res. (2013) 57:335–53. 10.1007/s12026-013-8466-z 24293007

[24] Zhang Y Garcia-Ibanez L Toellner KM. Regulation of germinal center B-cell differentiation. Immunol Rev. (2016) 270:8–19. 10.1111/imr.12396 26864101 PMC4755139

[25] Loken MR Shah VO Hollander Z Civin CI. Flow cytometric analysis of normal B lymphoid development. Pathol Immunopathol Res. (1988) 7:357–70. 10.1159/000157129 3266012

[26] Wang M Li Z Peng Y Fang J Fang T Wu J. Identification of immune cells and mrna associated with prognosis of gastric cancer. BMC Cancer. (2020) 20:206. 10.1186/s12885-020-6702-1 32164594 PMC7068972

[27] Colvin KL Cripe PJ Ivy DD Stenmark KR Yeager ME. Bronchus-associated lymphoid tissue in pulmonary hypertension produces pathologic autoantibodies. Am J Respir Crit Care Med. (2013) 188:1126–36. 10.1164/rccm.201302-0403OC 24093638 PMC3863738

[28] Mansueto G Di Napoli M Campobasso CP Slevin M. Pulmonary arterial hypertension (Pah) from autopsy study: T-Cells, B-cells and mastocytes detection as morphological evidence of immunologically mediated pathogenesis. Pathol Res Pract. (2021) 225:153552. 10.1016/j.prp.2021.153552 34352438

[29] Larsen KO Yndestad A Sjaastad I Loberg EM Goverud IL Halvorsen B. Lack of Ccr7 induces pulmonary hypertension involving perivascular leukocyte infiltration and inflammation. Am J Physiol Lung Cell Mol Physiol. (2011) 301:L50–9. 10.1152/ajplung.00048.2010 21498626

[30] Zamanian RT Badesch D Chung L Domsic RT Medsger T Pinckney A. Safety and efficacy of B-cell depletion with rituximab for the treatment of systemic sclerosis-associated pulmonary arterial hypertension: a multicenter, double-blind, randomized, placebo-controlled trial. Am J Respir Crit Care Med. (2021) 204:209–21. 10.1164/rccm.202009-3481OC 33651671 PMC8650794

[31] Hennigan S Channick RN Silverman GJ. Rituximab treatment of pulmonary arterial hypertension associated with systemic lupus erythematosus: a case report. Lupus. (2008) 17:754–6. 10.1177/0961203307087610 18625655

[32] Wang Y Liu J Burrows PD Wang JY B. Cell Development and maturation. Adv Exp Med Biol. (2020) 1254:1–22. 10.1007/978-981-15-3532-1_1 32323265

[33] Warnatz K Salzer U Rizzi M Fischer B Gutenberger S Böhm J. B-cell activating factor receptor deficiency is associated with an adult-onset antibody deficiency syndrome in humans. Proc Natl Acad Sci U S A. (2009) 106:13945–50. 10.1073/pnas.0903543106 19666484 PMC2722504

[34] Sasaki Y Casola S Kutok JL Rajewsky K Schmidt-Supprian M. Tnf family member B cell-activating factor (Baff) receptor-dependent and -independent roles for baff in B cell physiology. J Immunol. (2004) 173:2245–52. 10.4049/jimmunol.173.4.2245 15294936

[35] Stuber E Strober W. The T cell-B cell interaction Via Ox40-Ox40l is necessary for the t cell-dependent humoral immune response. J Exp Med. (1996) 183:979–89. 10.1084/jem.183.3.979 8642301 PMC2192367

[36] Cortini A Ellinghaus U Malik TH Cunninghame Graham DS Botto M Vyse TJ. Cell Ox40l supports T follicular helper cell development and contributes to sle pathogenesis. Ann Rheum Dis. (2017) 76:2095–103. 10.1136/annrheumdis-2017-211499 28818832 PMC5705841

[37] Wei H Wang JY. Role of polymeric immunoglobulin receptor in Iga and Igm transcytosis. Int J Mol Sci. (2021) 22:2284. 10.3390/ijms22052284 33668983 PMC7956327

[38] Shu T Xing Y Wang J. Autoimmunity in pulmonary arterial hypertension: evidence for local immunoglobulin production. Front Cardiovasc Med. (2021) 8:680109. 10.3389/fcvm.2021.680109 34621794 PMC8490641

[39] Matsushita T. Regulatory and effector B cells: friends or foes? J Dermatol Sci. (2019) 93:2–7. 10.1016/j.jdermsci.2018.11.008 30514664

[40] Corneth OBJ Klein Wolterink RGJ Hendriks RW. Btk signaling in B cell differentiation and autoimmunity. Curr Top Microbiol Immunol. (2016) 393:67–105. 10.1007/82_2015_478 26341110

[41] Kil LP de Bruijn MJ van Nimwegen M Corneth OB van Hamburg JP Dingjan GM. Btk levels set the threshold for B-Cell activation and negative selection of autoreactive B cells in mice. Blood. (2012) 119:3744–56. 10.1182/blood-2011-12-397919 22383797

[42] Heukels P Corneth OBJ van Uden D van Hulst JAC van den Toorn LM van den Bosch AE. Loss of immune homeostasis in patients with idiopathic pulmonary arterial hypertension. Thorax. (2021). 10.1136/thoraxjnl-2020-215460 33963088 PMC8606455

[43] Gómez Hernández G Morell M Alarcón-Riquelme ME. The role of Bank1 in B cell signaling and disease. Cells. (2021) 10:1184. 10.3390/cells10051184 34066164 PMC8151866

[44] Ramírez-Bello J Jiménez-Morales S Montufar-Robles I Fragoso JM Barbosa-Cobos RE Saavedra MA. Blk and Bank1 polymorphisms and interactions are associated in mexican patients with systemic lupus erythematosus. Inflamm Res. (2019) 68:705–13. 10.1007/s00011-019-01253-9 31134304

[45] Ramírez-Bello J Fragoso JM Alemán-Ávila I Jiménez-Morales S Campos-Parra AD Barbosa-Cobos RE. Association of Blk and Bank1 polymorphisms and interactions with rheumatoid arthritis in a latin-american population. Front Genet. (2020) 11:58. 10.3389/fgene.2020.00058 32153635 PMC7045059

[46] Rueda B Gourh P Broen J Agarwal SK Simeon C Ortego-Centeno N. Bank1 functional variants are associated with susceptibility to diffuse systemic sclerosis in caucasians. Ann Rheum Dis. (2010) 69:700–5. 10.1136/ard.2009.118174 19815934 PMC2975737

[47] Montúfar-Robles I Lara-García S Barbosa-Cobos RE Vargas-Alarcón G Hernández-Molina G Fragoso JM. Blk and Bank1 variants and interactions are associated with susceptibility for primary sjögren's syndrome and with some clinical features. Cell Immunol. (2021) 363:104320. 10.1016/j.cellimm.2021.104320 33756160

[48] Le Berre L Chesneau M Danger R Dubois F Chaussabel D Garand M. Connection of Bank1, tolerance, regulatory B cells, and apoptosis: perspectives of a reductionist investigation. Front Immunol. (2021) 12:589786. 10.3389/fimmu.2021.589786 33815360 PMC8015775

[49] Yang J Ren J Yang Y Sun J Zhou X Zheng S. Bank1 Alters B cell responses and influences the interactions between B cells and induced T regulatory cells in mice with collagen-induced arthritis. Arthritis Res Ther. (2018) 20:9. 10.1186/s13075-017-1503-x 29370826 PMC5785884

[50] Aiba Y Yamazaki T Okada T Gotoh K Sanjo H Ogata M. Bank negatively regulates akt activation and subsequent B cell responses. Immunity. (2006) 24:259–68. 10.1016/j.immuni.2006.01.002 16546095

[51] Costa RH Kalinichenko VV Lim L. Transcription factors in mouse lung development and function. Am J Physiol Lung Cell Mol Physiol. (2001) 280:L823–38. 10.1152/ajplung.2001.280.5.L823 11290504

[52] Pradhan A Dunn A Ustiyan V Bolte C Wang G Whitsett JA. The S52f Foxf1 mutation inhibits Stat3 signaling and causes alveolar capillary dysplasia. Am J Respir Crit Care Med. (2019) 200:1045–56. 10.1164/rccm.201810-1897OC 31199666 PMC6794119

[53] Karolak JA Gambin T Szafranski P Maywald RL Popek E Heaney JD. Perturbation of semaphorin and vegf signaling in acdmpv lungs due to Foxf1 deficiency. Respir Res. (2021) 22:212. 10.1186/s12931-021-01797-7 34315444 PMC8314029

[54] Ren X Ustiyan V Pradhan A Cai Y Havrilak JA Bolte CS. Foxf1 transcription factor is required for formation of embryonic vasculature by regulating vegf signaling in endothelial cells. Circ Res. (2014) 115:709–20. 10.1161/CIRCRESAHA.115.304382 25091710 PMC4810682

[55] Stankiewicz P Sen P Bhatt SS Storer M Xia Z Bejjani BA. Genomic and genic deletions of the fox gene cluster on 16q241 and inactivating mutations of Foxf1 cause alveolar capillary dysplasia and other malformations. Am J Hum Genet. (2009) 84:780–91. 10.1016/j.ajhg.2009.05.005 19500772 PMC2694971

[56] Cai Y Bolte C Le T Goda C Xu Y Kalin TV. Foxf1 maintains endothelial barrier function and prevents edema after lung injury. Sci Signal. (2016) 9:ra40. 10.1126/scisignal.aad1899 27095594

[57] Slot E Edel G Cutz E van Heijst A Post M Schnater M. Alveolar capillary dysplasia with misalignment of the pulmonary veins: clinical, histological, and genetic aspects. Pulm Circ. (2018) 8:2045894018795143. 10.1177/2045894018795143 30058937 PMC6108021

[58] Agarwal M Kumar P Mathew SJ. The Groucho/Transducin-like enhancer of split protein family in animal development. IUBMB Life. (2015) 67:472–81. 10.1002/iub.1395 26172616 PMC7015701

[59] Ramasamy S Saez B Mukhopadhyay S Ding D Ahmed AM Chen X. Tle1 tumor suppressor negatively regulates inflammation in vivo and modulates Nf-? b inflammatory pathway. Proc Natl Acad Sci USA. (2016) 113:1871–6. 10.1073/pnas.1511380113 26831087 PMC4763742

[60] Chen W Zheng D Mou T Pu J Dai J Huang Z. Tle1 attenuates hepatic ischemia/reperfusion injury by suppressing Nod2/Nf-Kb signaling. Biosci Biotechnol Biochem. (2020) 84:1176–82. 10.1080/09168451.2020.1735928 32114961

[61] Nimmo ER Stevens C Phillips AM Smith A Drummond HE Noble CL. Tle1 modifies the effects of Nod2 in the pathogenesis of Crohn's disease. Gastroenterology. (2011) 141:972-81. 10.1053/j.gastro.2011.05.043 21699783

[62] Eklow C Makrygiannakis D Backdahl L Padyukov L Ulfgren AK Lorentzen JC. Cellular distribution of the C-Type Ii lectin dendritic cell immunoreceptor (Dcir) and its expression in the rheumatic joint: identification of a subpopulation of Dcir+ T cells. Ann Rheum Dis. (2008) 67:1742–9. 10.1136/ard.2007.076976 18250113

[63] Kostarnoy AV Gancheva PG Lepenies B Tukhvatulin AI Dzharullaeva AS Polyakov NB. Receptor mincle promotes skin allergies and is capable of recognizing cholesterol sulfate. Proc Natl Acad Sci USA. (2017) 114:E2758–e65. 10.1073/pnas.1611665114 28292894 PMC5380039

