# Supplementary figures and images for: Weighted gene co-expression network analysis identifies dysregulated B-cell receptor signaling pathway and novel genes in pulmonary arterial hypertension
Source: Front Cardiovasc Med. 2022 Oct 6;9:909399. doi: 10.3389/fcvm.2022.909399 (PMC9583267; doi:10.3389/fcvm.2022.909399)

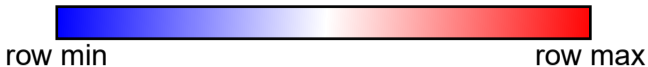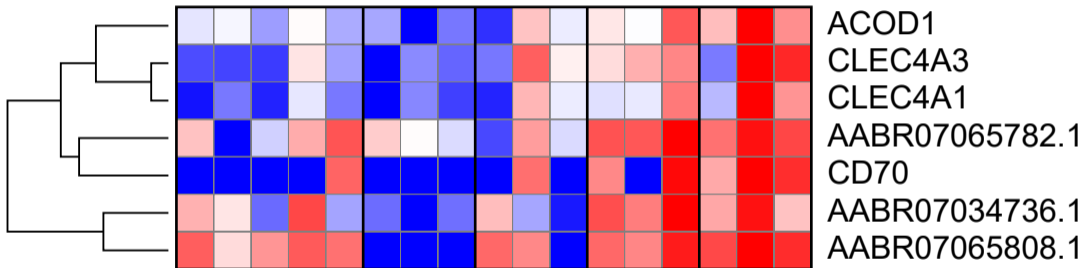

Weeks  
(MCT-treatment)

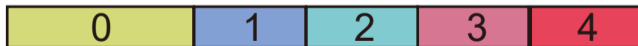

Supplement: Supplementary Figure 1 — Heatmap of seven hub genes in the dark red module. [file Data_Sheet_1.PDF]

row min

row max

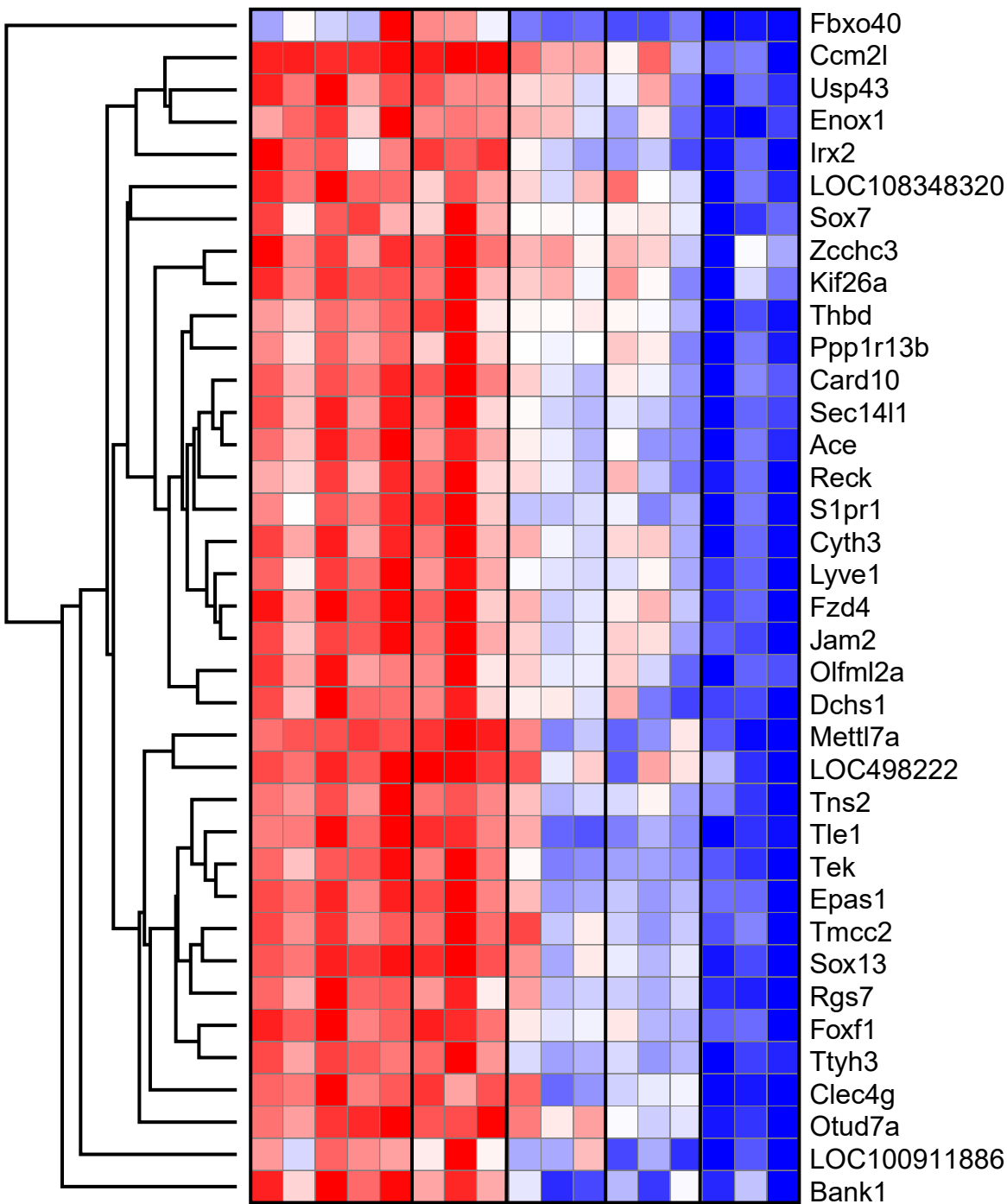

Weeks

0

1

2

3

4

(MCT-treatment)

Supplement: Supplementary Figure 2 — Heatmap of 37 hub genes in the blue module. [file Data_Sheet_2.PDF]
